# Basidiobolomycosis: Unusual Cause of Colonic Perforation

**DOI:** 10.7759/cureus.75318

**Published:** 2024-12-08

**Authors:** Mousa Mobarki, Nuraddin Alhakami, Maqsood Ahmad, Wagih Mommtaz Ghannam, Nisreen Mosaad, Shadi Hakami, Mansour Adawi, Fathi Ahmed Daifallah Fathi, Hamoud Al Amir, Mohammed Alsharif, Mohammed Majrashi, Mohammed Alharbi, Adeeb Salem, Mohammed Assiri, Abdulrahman A Muhajir, Abdulaziz H Alhazmi

**Affiliations:** 1 Department of Basic Medical Sciences (Pathology), Faculty of Medicine, Jazan University, Jazan, SAU; 2 Department of General Surgery, Armed Forces Hospital, Jazan, SAU; 3 Department of Histopathology, Jazan Regional Laboratory, Jazan Health Cluster, Jazan, SAU

**Keywords:** basidiobolomycosis, colon, fungus, gastrointestinal, perforation

## Abstract

Basidiobolomycosis is a rare fungal infection that is triggered by the environmental saprophyte *Basidiobolus ranarum*. Basidiobolomycosis usually presents as an infection beneath the skin and seldom impacts the digestive system. There is no clear clinical presentation, and the majority of initial cases are misdiagnosed. We describe a 68-year-old man who arrived at the emergency room with acute abdomen and shock with air under the diaphragm and needed urgent laparotomy with colonic resection. The patient, however, died from severe sepsis.

## Introduction

Basidiobolomycosis is a rare infection caused by the fungus *Basidiobolus ranarum*, an environmental saprophyte belonging to the class Zygomycetes and order Entomophthorales, found worldwide. The infection is typically subcutaneous and transmitted through traumatic inoculation [1].

Gastrointestinal basidiobolomycosis (GIB) is an uncommon manifestation of *Basidiobolus *infection. The first documented case of GIB was reported in Nigeria in 1964 in a six-year-old boy [2]. This form of the disease often affects the liver, colon, and small intestine, and can sometimes spread to the biliary tract and pancreas [3-5]. Many reported cases have originated from the Middle East, with countries like Iran, Saudi Arabia, and Kuwait being prominent sources [5]. Saudi Arabia, in particular, has a growing number of documented GIB cases [5]. Recently, Ghazwani et al. published a series of 25 GIB cases from the southwestern region of the country, highlighting multiple risk factors, such as exposure to insect bites, frogs, bats, and geckos, and emphasizing minor trauma, local inoculation as possible modes of transmission [5].

Due to its rarity, initial clinical suspicion of GIB is challenging, as there is no definitive clinical presentation, and cases are sometimes managed without a confirmed diagnosis [5]. The clinical and radiological features of GIB frequently mimic those of neoplastic or inflammatory bowel diseases, complicating early identification [4-5]. Diagnosis primarily relies on histological findings, including granulomatous inflammation, dense eosinophilic infiltrates, and the presence of fungal structures [5]. While microbiological culture confirmation is ideal, it is often impractical, as diagnoses are frequently made retrospectively. Although various complications of GIB have been documented, colonic perforation as a severe outcome remains underreported [5].

In this case report, we describe the clinicopathologic features of a patient diagnosed with GIB who succumbed to colonic perforation just two days after the diagnosis was confirmed. This work aims to deepen the understanding of basidiobolomycosis, highlight its clinical manifestations and complications, and discuss the challenges associated with its diagnosis and treatment.

## Case presentation

A 68-year-old male with a history of poorly controlled diabetes mellitus (DM) presented to the emergency department with abdominal pain persisting for five days, accompanied by vomiting and abdominal distension that began one day prior. The patient had been diagnosed with gastrointestinal basidiobolomycosis affecting the sigmoid colon two months earlier. The diagnosis was confirmed through an image-guided biopsy performed by interventional radiology, which identified characteristic fungal structures and since the diagnosis, he had been on a daily regimen of voriconazole, an antifungal medication. The physical examination of the current episode indicated that the patient was conscious, alert, and oriented. Vital signs showed a pulse of 90 beats per minute, blood pressure of 101/69, and temperature of 36.9°C with an oxygen saturation of 99% on room air. Abdominal examination revealed distention with generalized tenderness. Laboratory findings upon admission are summarized in Table 1.

**Table 1:** Laboratory parameters for the patient upon admission. RPS: random plasma sugar, (VBG) pH: venous blood gas pH, CO_2_: carbon dioxide, PO_2_: oxygen partial pressure, HCO_3_: bicarbonate.

Parameter	Value	Normal range	Unit
RPS	196	70-140 mg/dL	mg/dL
(VBG) pH	7.21	7.35-7.45	(Unitless)
CO_2_	31	35-45 mmHg	mmHg
PO_2_	40	75-100 mmHg	mmHg
HCO_3_	13	22-28 mEq/L	mEq/L
Lactate	8.8	0.5-2.2 mmol/L	mmol/L

**Figure 1:**
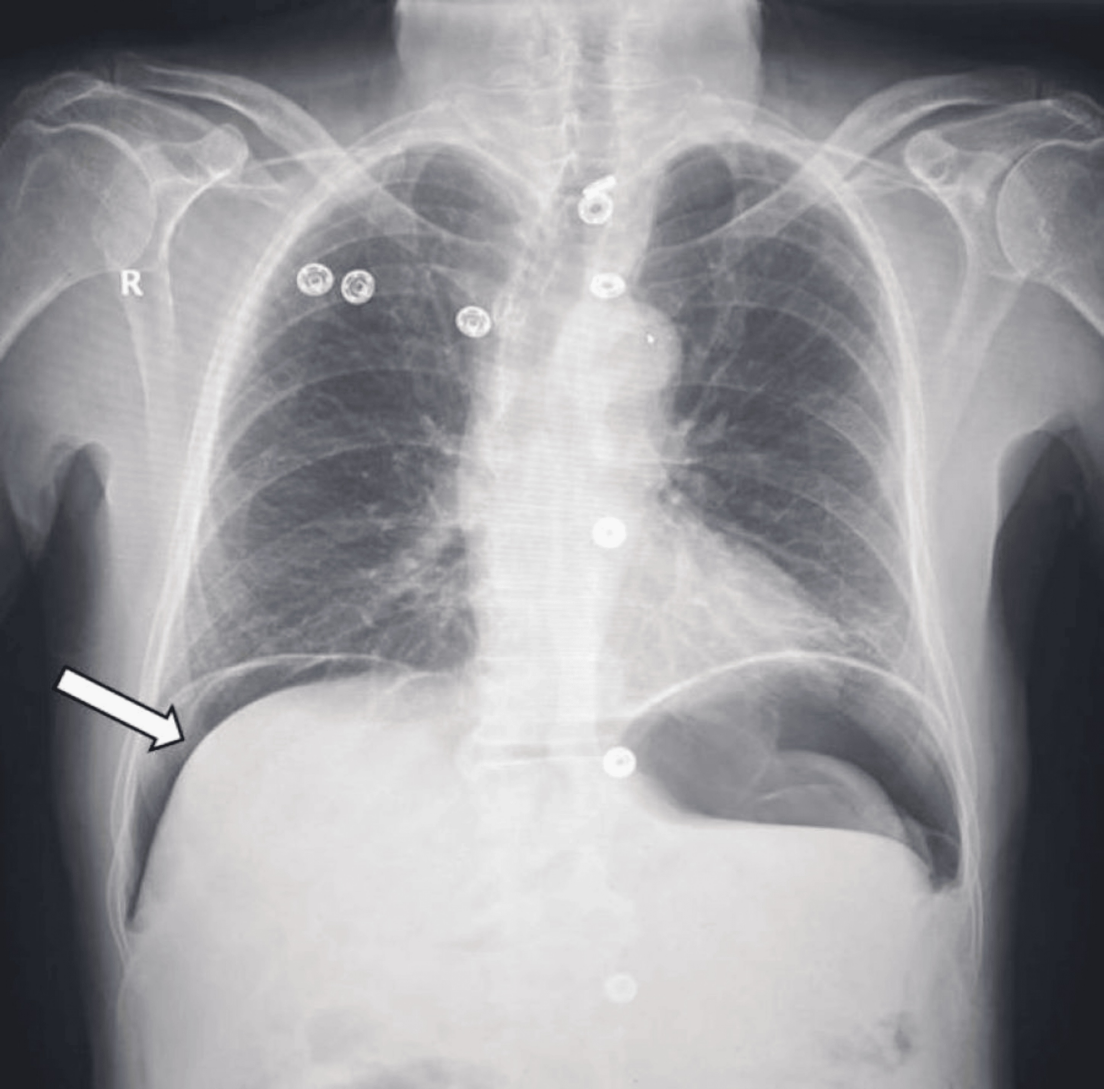
Erect abdominal X-ray showing air under diaphragm (pneumoperitoneum, arrow).

An erect abdominal X-ray demonstrated the presence of free air under the diaphragm, consistent with a perforated viscus (Figure 1).

**Figure 2:**
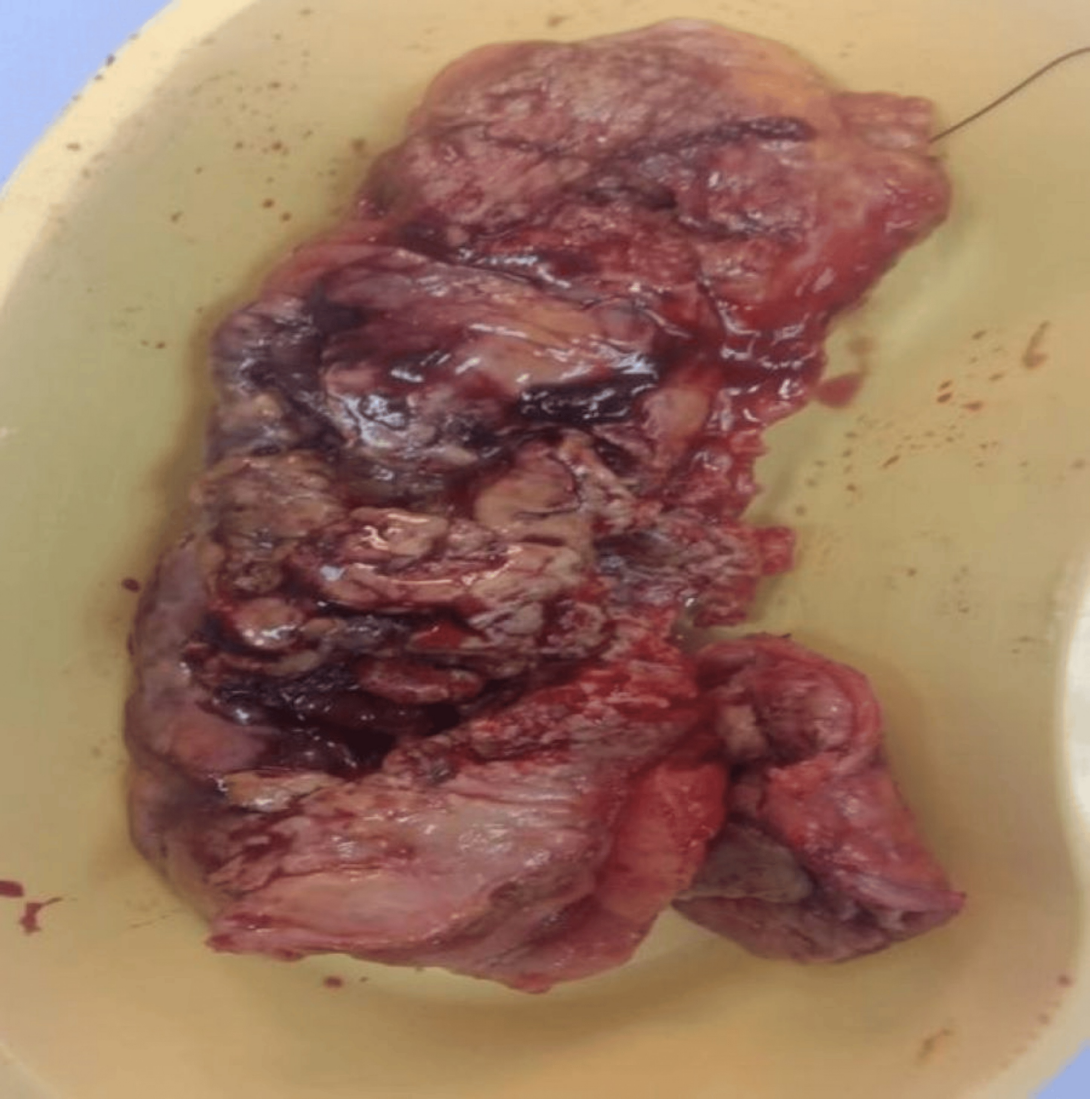
Macroscopic image of sigmoid colon with fungating mass causing secondary perforation.

The intervention was based on the diagnosis of perforated viscus. The patient was taken for exploratory laparotomy which revealed pus in the peritoneal cavity and mass in the sigmoid colon with perforation proximal to the mass, descending colon was resected with the Hartmann procedure, and then the patient shifted to ICU (Figure 2). Despite aggressive resuscitation and intensive care management, the patient developed septic shock and he was declared dead on the second postoperative day.

**Figure 3:**
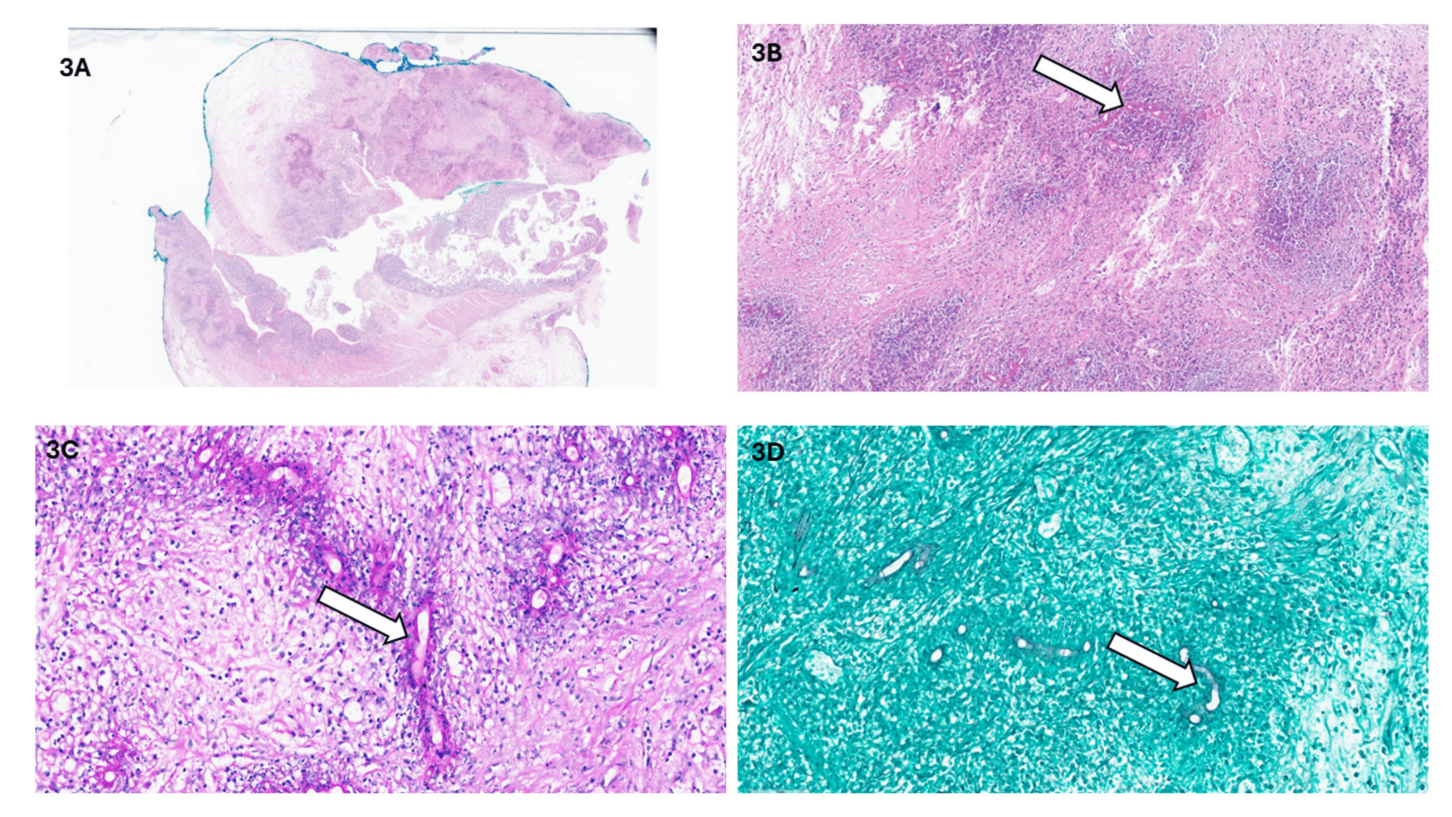
A: Low power image showing a diffuse transmural inflammatory process affecting the upper left fragment with perforation in comparison to the lower left fragment with viable lining mucosa. B: Microscopic image diffuse active granulomatous eosinophilic inflammation with thin broad septate hyphea fungal hyphea with splendore-hoeppli phenomenon (arrow). C: PAS (Periodic Acid Schiff) special stain highlighting the fungal hyphae (arrow). D: GMS (Grocott's methenamine silver) special stain highlighting the fungal hyphae (arrow).

The resected colonic specimen measured 14 x 4 x 3.5 cm, exhibiting multiple perforations and a markedly thickened wall with an irregular, rough external surface. Cut sections showed a firm, perforated colonic wall with a mural thickness of up to 1.5 cm. Histopathological examination revealed diffuse, active granulomatous eosinophilic colitis with evidence of fungal hyphae characterized by thin, broad-based, septated structures surrounded by a Splendore-Hoeppli phenomenon. These fungal elements were highlighted using Periodic Acid-Schiff (PAS) and Gomori Methenamine Silver (GMS) special stains (Figures 3A-3D). Surgical margins showed no evidence of malignancy observed in the examined tissue sections.

## Discussion

Gastrointestinal basidiobolomycosis (GIB) is a rare, yet potentially fatal fungal infection caused by *Basidiobolus ranarum*, an environmental saprophytic fungus found worldwide in soil, decaying organic matter, and within the gastrointestinal tracts of amphibians, reptiles, fish, and insectivorous bats [5]. Due to its nonspecific clinical presentation, GIB is often misdiagnosed as inflammatory bowel disease or gastrointestinal malignancy [4-5]. In Saudi Arabia, several cases of GIB have been reported, predominantly affecting male patients residing in rural or mountainous regions [5]. Common symptoms include poor appetite, abdominal pain, a palpable abdominal mass, weight loss, and general malaise. The nonspecific nature of these symptoms often leads to delays in diagnosis, increasing the risk of severe complications, morbidity, and mortality [5-7].

The case presented here demonstrated histopathological, laboratory, and imaging findings consistent with previously documented reports. As GIB is life-threatening, its management requires careful and timely intervention [5]. However, the literature lacks consensus on a definitive treatment regimen. Current strategies typically involve prolonged antifungal therapy, either as monotherapy or combined with surgical resection and debridement of infected tissue [2,4,5].

The challenges we encountered in this case were multifaceted. Although the patient had presented two months earlier, received a successful diagnosis of GIB, and was treated with voriconazole, a drug known for its efficacy in such infections, the patient’s later presentation indicated a failure of the initial treatment. Unfortunately, our current methods could not definitively identify whether this treatment failure was due to poor adherence to the prescribed voriconazole dosage or an unreported resistance of Basidiobolus to the antifungal agent. Previous reports have highlighted that effective management of GIB often requires prolonged antifungal therapy, potentially extending for up to 12 months [2,5]. This extended treatment period necessitates regular follow-up to ensure strict adherence to the regimen, as nonadherence can lead to subtherapeutic dosing and subsequent treatment failure [6]. Additionally, treatment failure may occur if Basidiobolus develops resistance to the antifungal medication used [6-9]. Given the lack of consensus on the optimal treatment and dosage for GIB, data on resistance patterns and effective management strategies remain scarce. This experience indicates the necessity for rigorous adherence monitoring and further research to establish standardized, evidence-based treatment protocols for GIB to improve patient outcomes.

Another significant challenge we face with GIB is the rare but severe complication of colonic perforation [8,9]. This complication tends to manifest at an advanced stage when *Basidiobolus* has disseminated widely in the abdominal cavity, significantly increasing morbidity and mortality risks. Colonic perforation is infrequently reported, and available literature often describes late-stage, complex presentations. For instance, a case from the United Arab Emirates detailed the experience of a 20-month-old boy diagnosed with colonic basidiobolomycosis [9]. The diagnosis was primarily based on histopathological findings from bowel biopsies, which revealed an intense inflammatory reaction characterized by sheets of eosinophils, giant cells, and granulomas with central necrosis. The clinicians initially missed the microbiological evaluation, which delayed the definitive diagnosis and indicated the critical importance of comprehensive diagnostics for earlier detection. As the child's condition worsened, marked by severe abdominal distension and a palpable colonic mass, an exploratory laparotomy revealed an extensive, heterogeneous mass. This mass originated from the left and transverse colon, involved the splenic flexure, and adhered to the sigmoid colon. Microbiological cultures confirmed the presence of *Basidiobolus ranarum*. Notably, the fungal pathogen displayed resistance to voriconazole but remained sensitive to itraconazole and amphotericin B. Administering a combination of itraconazole, and amphotericin B led to a favorable outcome for the patient, highlighting the importance of susceptibility testing in guiding effective antifungal therapy [6,8,9]. The current understanding of optimal treatment strategies for colonic basidiobolomycosis is still evolving, as evidenced by the limited number of cases in the literature [5,6,9]. Surgical resection, coupled with prolonged antifungal therapy, appears to be the most effective therapeutic approach, often resulting in complete disease resolution [5]. This case emphasizes the need for early recognition, thorough diagnostic workup, and individualized treatment plans to manage this potentially life-threatening infection successfully.

The main limitations of this study include its retrospective design, which restricts the ability to draw causal inferences, and the absence of comprehensive clinical data, such as patient adherence to antifungal therapy and potential resistance patterns. Additionally, the lack of standardized treatment protocols for GIB and the limited number of reported cases makes it difficult to generalize findings.

## Conclusions

GIB is a rare but serious fungal infection caused by *Basidiobolus ranarum*, often misdiagnosed due to nonspecific symptoms like abdominal pain and weight loss. In Saudi Arabia, cases are more common in rural males, and delayed diagnosis can increase mortality. Histopathological evidence is key for primary identification and microbiology work-up seems essential fora confirmed diagnosis and better management. Treatment usually involves prolonged antifungal therapy and may require surgery. However, challenges like treatment adherence and drug resistance complicate management. Cases highlight the need for susceptibility testing and consistent follow-up, with a combined approach of surgery and extended antifungal therapy proving most effective. More research is needed to establish standardized protocols.
